# Massive rectal bleeding from acquired jejunal diverticula

**DOI:** 10.1186/1749-7922-6-17

**Published:** 2011-05-13

**Authors:** Sheraz Yaqub, Birte V Evensen, Kristin Kjellevold

**Affiliations:** 1 Department of Gastrointestinal Surgery, Akershus University Hospital, 1478 Lørenskog, Norway; 2 Department of Radiology, Akershus University Hospital, 1478 Lørenskog, Norway

## Abstract

Small bowel diverticulosis is an uncommon and often asymptomatic condition that is sporadically observed during radiographic examination or laparotomy. Although it is frequently seen in duodenum, jejunal and ileal locations are very rare. The majority of patients with jejunal diverticula have no symptoms. However, they can present with a number of acute and emergent complications with a high rate of mortality. Bleeding from jejunal diverticula occurs in less than 3% - 8% of patients and often present as fresh rectal haemorrhage. This can confuse the clinician since a bleeding source in colon is far more common. We report a patient with acute massive rectal bleeding. Abdominal CT angiography demonstrated a jejunal diverticulum as the bleeding source and the patient underwent resection of the affected segment. She has since remained free of gastrointestinal bleeding.

Although jejunal diverticulosis is rare, it is an important differential diagnosis for patients with gastrointestinal haemorrhage of unknown origin as it may cause extensive rectal bleeding. Abdominal CT angiography can localize the bleeding source and resection of the affected bowel and primary anastomosis is the treatment of choice.

## Background

**Figure 1:**
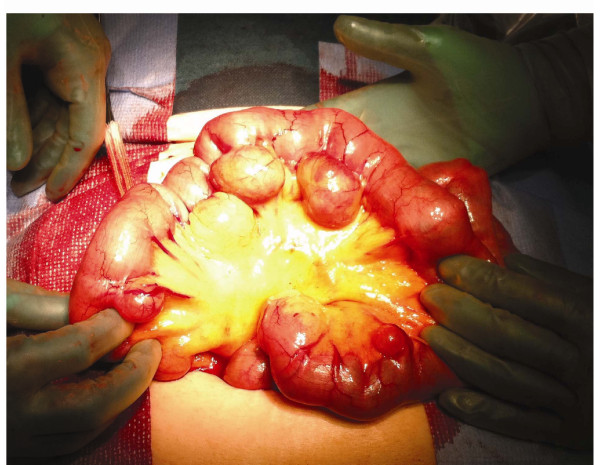
**Jejunal diverticula**. Intraoperative photograph demonstrating multiple jejunal diverticula. Note that the diverticula arise at the mesenteric border.

Acquired diverticula of the jejunum and ileum are an uncommon entity, with a reported prevalence of 0.3% - 1.9% on small bowel studies and 0.3% - 1.3% at autopsy studies [1-4]. About 80% of diverticula occur in the jejunum and two-thirds of patients have multiple diverticula, but the number decreases distally with a solitary diverticulum commonly found in the ileum [5]. The highest incidence of jejunal diverticula is in the elderly, occurring during the sixth and seventh decades of life, and is thought to be more common in males. Jejunoileal diverticula are acquired false diverticula as they lack a true muscular wall and are thin and fragile. They are pulsion diverticula thought to be the result of intestinal dyskinesia leading to high intraluminal pressure. This results in herniation of mucosa and submucosa through the weakest site of the muscularis, which is where blood vessels penetrate into the bowel wall. This explains the common location of these diverticula at the mesenteric side of the bowel (Figure 1).

Malabsorption due to bacterial overgrowth is the major clinical manifestation of jejunoileal diverticula. Inflammation, perforation, and bleeding are far less common than in colon diverticula. The most common lesions leading to small bowel bleeding are tumors, arteriovenous malformations, and inflammatory bowel disease. Massive gastrointestinal haemorrhage from jejunal diverticula is extremely rare. However, it has been associated with high mortality rate caused by delayed diagnosis.

We report a case of massive rectal haemorrhage from a jejunal diverticulum and discuss diagnostic evaluations and treatment options.

## Case presentation

A 74-year-old female was admitted to our hospital after an episode of massive rectal bleeding. Her past medical history was significant for hypertension and non-insulin dependent diabetes mellitus. In addition to anti-hypertensive and anti-diabetic drugs, she was taking aspirin 75 mg daily. There was no previous history of gastrointestinal haemorrhage. The bleeding started at home some hours before admission. Upon arrival at the emergency room, she was awake and alert. On physical examination, the blood pressure was 130/80 mmHg, and the pulse was 60 beats/min. The abdomen was soft, non-distended and non-tender. On rectal examination, old blood on the glove was noticed. The initial haemoglobin level was 10.8 g/dL, trombocytes 186 x10^9^/L, and C-reactive protein <5 mg/L. The bleeding appeared to have ceased and the patient was considered haemodynamically stable. She had no more episodes of rectal bleeding during the night or the next morning and was discharged with an urgent appointment for outpatient workup with colonoscopy.

**Figure 2:**
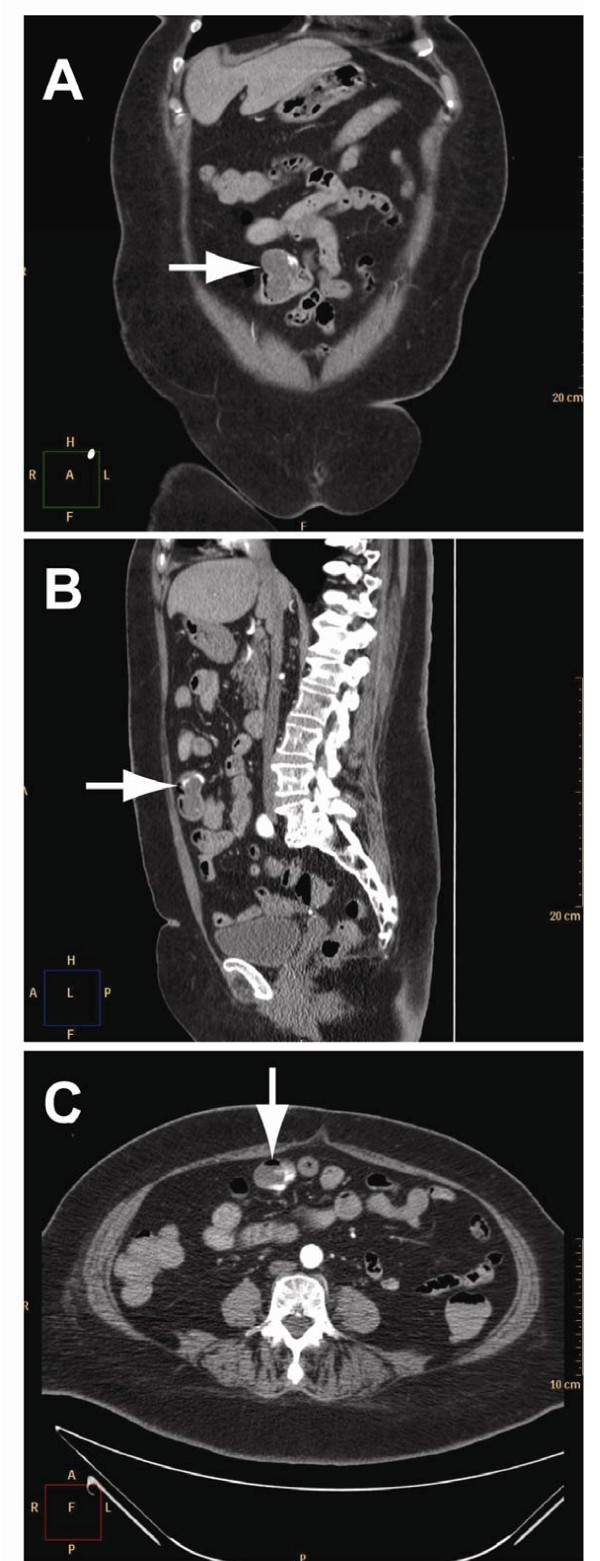
**Abdominal computed tomography (CT) angiography in arterial phase**. **A**, Coronal abdominal CT demonstrating contrast extravasation in small intestine diverticulum, diagnostic for bleeding (white arrow). **B**, Jejunal diverticulum with bleeding seen on sagittal abdominal CT (white arrow). **C**, The bleeding in jejunal diverticulum demonstrated on axial abdominal CT (white arrow).

The rectal bleeding recurred at home 10 hours after discharge. She had an episode of syncope and passed red blood per rectum. She was urgently brought back to the emergency department at our hospital. On physical examination she was pale and diaphoretic, with a blood pressure of 105/53 mmHg and a pulse rate of 105 beats/min. The abdomen was non-tender and fresh blood was observed in the rectum. The haemoglobin level was 8.4 g/dL, haematocrit value was 25%, and trombocytes 122 x10^9^/L. To localize the source of bleeding, the patient underwent acute abdominal CT angiography, which revealed bleeding in a jejunal diverticulum (Figure 2). The abdominal CT also demonstrated multiple colonic diverticula, but did not show any bleeding in the colon. Immediately after the diagnosis of jejunal diverticular haemorrhage was made, the patient was brought to the operating room. At laparotomy, multiple large diverticula in a 30 cm segment of jejunum were confirmed, beginning 90 cm distal to the ligament of Treitz (Figure 1). Some smaller diverticula in distal jejunum were also registered. Systematic exploration of the abdomen revealed diverticulosis of the left colon, but no other lesions. In order to localize the exact bleeding site, an enterotomy proximal to the most proximal diverticulum was performed, and a gastroscope was introduced. Blood in the intestine at the level of the second diverticulum was found. The 30 cm segment of jejunum containing large diverticula was resected and a primary anastomosis performed. The patient was transfused with 4 units of packed red cells, 3 units of fresh frozen plasma, and 2 units of trombocytes. The postoperative course was uneventful and the patient was discharged on postoperative Day 5 with a haemoglobin level at 9.7 g/dL. Final pathology of the resected specimen confirmed multiple jejunal diverticula, but did not locate any ulcers. The patient had no further episodes of gastrointestinal bleeding, confirming that the bleeding source was in the jejunal diverticulum.

## Discussion

Jejunoileal diverticula were first time described by Soemmering in 1794 and Sir Astley Cooper in 1807 [6]. They are found at the mesenteric side of the small intestine where the arteries enter the intestine. Nearly 80% occur in the jejunum, approximately 15% in the ileum, and 5% in both [5]. Jejunal diverticulosis is a rare entity and the majority of patients have no symptoms. As a result, identification of the disorder can be quite difficult. However, it can present with a number of complications that require quick diagnosis and acute surgical care [7,8]. The reported complications of jejunal diverticulosis include haemorrhage, malabsorption, volvulus, diverticulitis, obstruction, abscess, and perforation, and occur in 10% - 30% of patients [1,7,8]. Colonic diverticula have a high association with the presence of jejunal diverticula [9]. The clinician should suspect small bowel diverticulosis if there is a history of colonic diverticula. CT scan can be helpful in diagnosis of jejunal diverticula and can differentiate them from other inflammatory conditions such as colon diverticulitis and appendicitis [10]. Our patient also had coexisting colonic diverticula which were initially suspected to be the source of bleeding.

Haemorrhage as a presenting symptom occurs in 3.4% - 8.1% of patients with this condition [3,11]. There have been less than 60 case reports in the English literature describing massive haemorrhage from jejunal diverticula [8]. Unfortunately, neither the history nor the physical examination are helpful in diagnosing jejunal diverticula. These patients often experience acute massive bleeding per rectum and most patients have had no previous gastrointestinal symptoms. Furthermore, the acute haemorrhage is likely to recur if the diagnosis of bleeding jejunal diverticula is missed at the initial presentation, as was the case with our patient.

**Figure 3:**
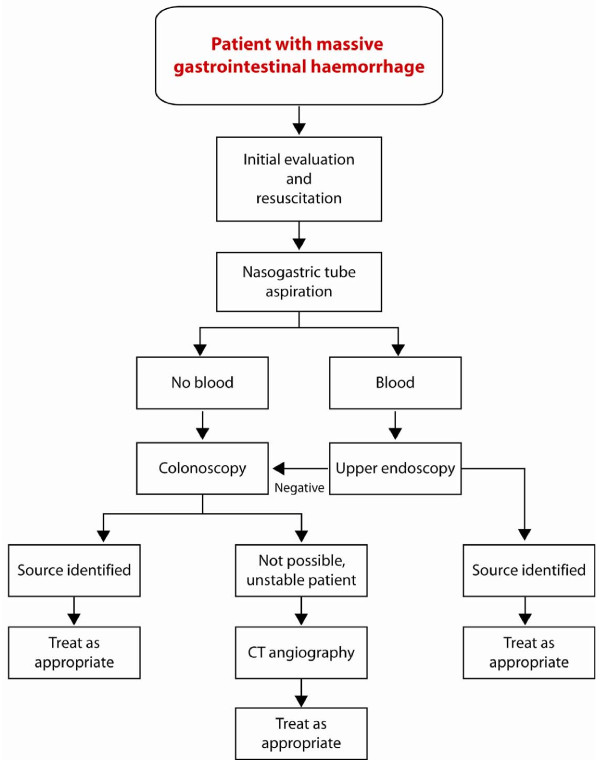
**Diagnostic approach to gastrointestinal bleeding**.

In patients with rectal bleeding, the diagnostic challenge is the location of the bleeding source. If the bleeding site is in the colon, it can usually be located by colonoscopy. However, it is often not easy due to poor visualization in unprepared colon and massive haemorrhage can obscure the bleeding site. If the bleeding source is in the small intestine it is often impossible to find it endoscopically, although there are some reports showing success with capsule endoscopy and double balloon endoscopy [12,13]. The utility of these examinations are however limited in emergency situations as in the presented case [14]. Non-invasive imaging with technetium-99m (Tc-99)-labelled red blood cell scintigraphy can be used to detect and localize gastrointestinal bleeding. It has been reported to have a sensitivity of 93% and specificity of 95% for detecting a bleeding site with bleeding rate as low as 0.2 mL/min [15]. However, Tc-99 scintigraphy has a false localization rate of approximately 22%, which limits its value as a diagnostic test [16]. Mesenteric angiography can detect bleeding rates greater than 0.5 mL/min and has the advantage of therapeutic intervention through transcatheter embolization, but it has a sensitivity of 40% - 86% [17]. Angiographic embolization has been successful in some cases, but carries the risk of ischemia [18]. Our diagnostic approach in the haemodynamically stable patients presenting with lower gastrointestinal haemorrhage is endoscopy. Upper and lower gastrointestinal endoscopy must be performed in all cases presenting with massive lower gastrointestinal bleeding. Finding of blood at certain segments can provide valuable information on the localization of the bleeding source. However, in patients with ongoing lower gastrointestinal bleeding or with negative or inconclusive endoscopy, the preferred diagnostic approach is abdominal CT angiography in attempt to localize the source of haemorrhage (Figure 3). A recent meta-analysis showed that CT angiography is a time-efficient, cost effective, and accurate tool in the diagnosis or exclusion of acute gastrointestinal bleeding [19]. Arterial phase CT angiography can depict active extravasation of contrast material into the intestinal lumen, a finding diagnostic of ongoing gastrointestinal bleeding. CT angiography can thereby pinpoint the location of the bleeding source, and direct further management [19,20].

Haemodynamically unstable patients with massive rectal haemorrhage should undergo emergency laparotomy [1]. Although the colon is the most likely source of extensive rectal bleeding in patients above 50 years of age, a high index of suspicion of a small intestinal site of bleeding should be maintained. It is mandatory to systematically inspect the small intestine, and owing to the mesenteric location of the diverticula, the intraoperative recognition can be facilitated by jejunal insufflations using manual compression [1]. If no small intestine diverticula are found, a subtotal colectomy is recommended [1]. When jejunal diverticula are identified as the bleeding source, either preoperatively or intraoperatively, partial resection of the involved segment of jejunum with primary anastomosis is the procedure of choice. A special challenge is in patients with multiple diverticula along the small intestine, where it is not possible to remove all of them. In such cases it is easy and safe to perform an intraoperative endoscopy through an enterotomy, which effectively can localize the bleeding source [21]. Another dilemma is that approximately 50% of patients with jejunal diverticula also have coexisting colonic diverticula. In such patients a preoperatively CT angiography can be helpful to pinpoint the bleeding source and thus avoid unnecessary colectomy. However, even when the preoperative studies implicate bleeding from colon, the finding of jejunal diverticula at laparotomy is justification for resection of the involved small intestine [22]. Failure to identify and remove jejunal diverticula may lead to continued bleeding after blind colectomy.

In our case, as in many others with bleeding from jejunal diverticulosis, pathologic examination of the resected bowel segment did not localize the bleeding site. We consider the immediate and long-term cassation of bleeding achieved by resection of the diverticula as a satisfactory confirmation of diagnosis of jejunal diverticular haemorrhage [23].

## Conclusion

Jejunoileal diverticulosis is an uncommon entity and a rare source of gastrointestinal haemorrhage. However, it should be considered in all patients with acute bleeding in the lower part of the gastrointestinal tract, especially in the elderly, because it may lead to life threatening complications and death. In case of massive ongoing rectal bleeding, CT angiography is an accurate, rapid, and non-invasive modality that may detect the bleeding site. If unstable or multiple jejunal diverticula, an intraoperative endoscopy can be performed safely via an enterotomy to localize the bleeding site. Surgical resection of the involved intestine and primary anastomosis is the treatment of choice.

## Consent

Written informed consent was obtained from the patient for publication of this Case report and any accompanying images. A copy of the written consent is available for review by the Editor-in-Chief of this journal.

## List of abbreviations

CT: Computed tomography.

## Competing interests

The authors declare that they have no competing interests.

## Authors' contributions

SY conducted the literature search, completed the chart review and authored the manuscript. KK provided input to the manuscript, edited the manuscript and operated the patient with SY. BVE provided the preoperative CT scan assessment and provided input to the manuscript. All authors read and approved the final manuscript.
